# Proceedings of a Meeting of the County Medical Societies of Northern Indiana

**Published:** 1866-04

**Authors:** C. R. Richmond, N. W. Black

**Affiliations:** Pres’t.; City of Kokomo; Sec’y.; City of Kokomo


### PROCEEDINGS OF A MEETING OF THE COUNTY MEDICAL SOCIETIES OF NORTHERN INDIANA.

CITY OF KOKOMO, Feb. 27, 1866.

According to previous arrangement, a number of physicians, members of the different county medical societies of Northern Indiana, met in the City of Kokomo, at 10 o'clock A.M., for the purpose of taking into consideration the propriety of a more thorough organization of the medical profession.

On motion, Dr. C. RICHMOND, of Howard Co., was chosen President, and Dr. N. W. BLACK, of Delaware Co., Secretary.

The following physicians presented their credentials as delegates:—Drs. L. B. Harvey and L. D. Waterman, of Marion Co.; Wm. Lomax and H. Charles, of Grant Co.; R. Winton and N. W. Black, of Delaware Co.; C. Richmond, L. Kern, and Wm. Scott, of Howard Co.; and M. M. Jones, of Tipton Co.

Dr. Wm. Lomax then stated the object of the meeting in a clear and impressive manner.

The Society was then entertained by very interesting remarks from Drs. L. B. Harvey and L. D. Waterman.

On motion, the Society then adjourned, to meet at 2 o'clock P.M.

2 P.M.—Society met, according to adjournment.

The President called the house to order, and the minutes of the previous session were read and approved.

The following members of the profession were admitted as delegates:—Drs. G. Lyons, J. E. Lyons, and R. A. Curran, of Huntington Co.; R. S. Blount, W. R. Winton, and J. L. Dicken, of Wabash Co.

On motion of Dr. R. A. Curran, a committee composed of one member from each county represented, was appointed to draft an address to the physicians of the State, urging the necessity of a more thorough organization of the medical profession.

The President then appointed the following members such committee:—Drs. Wm. Lomax, J. L. Dicken, R. Winton, R. A. Curran, L. B. Harvey, M. M. Jones, L. Kern, and N. S. Wickersham.

Dr. N. S. Wickersham was then admitted as a delegated member from Madison Co.

Dr. Wm. R. Winton then read an essay on medical organization, which was listened to with interest and received the applause of all present.

The Society then formally adjourned, to meet at 7 P.M.

7 P.M.—The President called the house to order, after which the Committee on Organization made their report, which, on motion, was accepted.

Dr. R. A. Curran moved to recommit the report for revision and correction, which motion was adopted.

On motion, Dr. Wm. Lomax was appointed a committee to have the address published; and that the expenses incurred in publishing and distributing the same shall be proportioned equally amongst the different societies represented.

On motion, it was ordered that 1000 copies be published and distributed throughout the State.

On motion, the Secretary was requested to have the proceedings of the meeting published in the *Western Lancet*, CHICAGO MEDICAL EXAMINER, *State Weekly Journal, and State Herald.*

On motion, the Society adjourned and repaired to a place where the physicians of Kokomo had served up a fine oyster supper, which was agreeably relished by the inner man, after which there were some spicy jokes passed, a hearty shake of the hands, a good-bye, etc., and as we left for our respective homes we felt the impression on our minds that our time had been most interestingly and profitably spent.

C. R. RICHMOND, M.D., *Pres't.*

N. W. BLACK, M.D., *Sec'y.*

